# The BET/BRD inhibitor JQ1 improves brain plasticity in WT and APP mice

**DOI:** 10.1038/tp.2017.202

**Published:** 2017-09-26

**Authors:** E Benito, B Ramachandran, H Schroeder, G Schmidt, H Urbanke, S Burkhardt, V Capece, C Dean, A Fischer

**Affiliations:** 1 Department for Epigenetics and Systems Medicine in Neurodegenerative Diseases, German Center for Neurodegenerative Diseases (DZNE) Göttingen, Göttingen, Germany; 2 Trans-synaptic Signaling Group, European Neuroscience Institute, Goettingen, Germany; 3 Department of General, Visceral and Pediatric Surgery, University Medical Center Göttingen, Göttingen, Germany; 4 Department of Psychiatry and Psychotherapy, University Medical Center Göttingen, Göttingen, Germany

## Abstract

Histone acetylation is essential for memory formation and its deregulation contributes to the pathogenesis of Alzheimer’s disease. Thus, targeting histone acetylation is discussed as a novel approach to treat dementia. The histone acetylation landscape is shaped by chromatin *writer* and *eraser* proteins, while *readers* link chromatin state to cellular function. Chromatin readers emerged novel drug targets in cancer research but little is known about the manipulation of *readers* in the adult brain. Here we tested the effect of JQ1—a small-molecule inhibitor of the chromatin readers BRD2, BRD3, BRD4 and BRDT—on brain function and show that JQ1 is able to enhance cognitive performance and long-term potentiation in wild-type animals and in a mouse model for Alzheimer’s disease. Systemic administration of JQ1 elicited a hippocampal gene expression program that is associated with ion channel activity, transcription and DNA repair. Our findings suggest that JQ1 could be used as a therapy against dementia and should be further tested in the context of learning and memory.

## Introduction

There is substantial evidence that histone acetylation plays a key role in memory consolidation and deregulated histone acetylation has been linked to neurodegenerative diseases such as Alzheimer’s disease (AD)^1, 2, 3^. The histone acetylation landscape is shaped by the counteracting activity of so-called ‘writer’ and ‘eraser’ proteins that add or remove acetyl groups from histone proteins.^4, 5^ Histone deacetylases (HDACs) that constitute part of the ‘eraser’ activity in particular have gained increasing interest, since HDAC inhibitors were shown to ameliorate disease phenotypes in models for various neurodegenerative diseases.^6, 7^ In contrast, there is only limited knowledge about the role of ‘chromatin readers’ that recognize combinatorial patters of histone modifications and thus provide the essential link between histone changes and corresponding changes in cellular function. The Bromodomain Extraterminal (BET) subfamily of chromatin readers is highly specific toward acetylated histone tails, with highest affinity for H4K12, and H4K5.^8, 9^ This is interesting, as BRD2 and BRD4 preferentially bind H4K12ac.^8, 9^ Moreover, H4K5ac and H4K12ac were specifically linked to memory function and age-associated memory impairment.^10, 11, 12^

In addition to their high target specificity, BETs have gained attention due to the recent development of very efficient small-molecule inhibitors that block their binding to acetylated residues.^13, 14^ JQ1 is a BET inhibitor with highest specificity for BRD4^14^ and it (or its derivative molecules) has shown beneficial effects in several disease models, including leukemia,^15, 16^ neuroblastoma,^17^ arthritis^18^ and pathological heart remodeling.^19^

However, the effect of JQ1 and other BET/BRD inhibitors in the adult brain is not well defined and the few data that exist does not allow for a defined conclusion.^20, 21, 22, 23, 24^ As such, a detailed investigation on the effects of JQ1 on memory function and synaptic plasticity in the context of neurodegenerative diseases is missing. Thus, we decided to test the effect of JQ1 on cognitive performance. We found that JQ1 improves associative memory and enhances spatial memory precision both in wild-type animals and in a mouse model of AD. We also carried out electrophysiological measurements and found that JQ1 enhances long-term potentiation (LTP). Gene expression profiling of the hippocampal CA1 region of JQ1-treated animals revealed functional networks associated with ion channel activity and transcription, and with additional DNA repair subnetworks in APP/PS1–21 animals. Our data provide evidence that JQ1 and related BET/BRD inhibitors may be suitable drugs to treat dementia.

## Materials and methods

### Animals

All experiments were carried out complying with local regulations under protocol G12/961. Ten-week-old C57Bl/6j males were purchased from Janvier (Le Genest St Isle, France) and allowed to habituate to the facility for at least 1 week before any handling or experiments began. APP/PS1–21 animals belonged to the Tg(Thy1-APPSw,Thy1-PSEN1*L166P)21Jckr line. Females (8–10 months old) were used for behavioral experiments. Animals were kept in a 12 h/12 h light/dark cycle and provided with food and water *ad libitum*. Animals were single-caged prior to behavioral testing.

### 3JQ1 injections and behavior

JQ1 was initially a gift from Dr Jay Bradner and later was purchased form ApexBio (Taufkirchen, Germany). JQ1 was dissolved at 50 mg ml^−1^ in DMSO and kept in frozen aliquots at −20 °C. The JQ1 injection solution was prepared fresh every day by mixing nine parts of 10% bega-cyclodextrin (Sigma, Taufkirchen, Germany) with one part of JQ1 as per the recommendation from the Bradner lab. Animals were given an intraperitoneal 50 mg kg^−1^ dose at the indicated times in the different experimental settings. For the open field test, animals were given daily JQ1 injections 1 week prior to the behavioral test. On the day of testing, animals were placed in a 50cm × 50cm gray box and their activity tracked and recorded automatically for 25 min with a TSE tracking system and using the proprietary software Videomot (Bad Homburg, Germany) (TSE). For fear-conditioning, mice were introduced in the conditioning chamber (Med Associates) for 3 min, after which they received a 2 s 0.5 mA footshock. Animals were then removed from the chamber and put back in their home cage for 24 h or 4 weeks prior to testing. On the testing day, animals were reintroduced into the conditioning context and freezing behavior was automatically recorded during 3 min using the proprietary software Video Freeze (Med Associates). For water maze experiments, animals were given four consecutive trials with randomized entry points in a round pool filled with opaque water. Each trial lasted 1 min. If the animals did not find the platform within this time, they were gently guided to the platform and allowed to stay on the platform for 15 s. Latency to reach the platform was recorded using TSE’s Videomot software. On probe test day, the platform was removed and the percentage of time spent in the target quadrant and percentage of time spent in the platform area were recorded over the course of 1 min. For rotarod experiments, animals were trained on two consecutive days at 5 r.p.m. and tested on day 3 with an acceleration from 5 to 40 r.p.m. over 240 s. The latency and r.p.m. to fall were recorded using TSE’s Rotarod Advanced system.

### Cannulation and intrahippocampal JQ1 injections

Animals that received intrahippocampal JQ1 injections were given metamizol in drinking water (3 ml l^−1^) 3 days prior to the operation and until 4 days post operation. Animals were anesthetized with ketamine/xylazine (80 and 10 mg kg^−1^, respectively) and were placed in a stereotaxic device (myNeuroLab, Wetzlar, Germany). Holes were drilled at anteroposterior −1.75 mm and mediolateral ±1 mm from bregma, and 2 mm long cannulae implanted and fixed with dental cement. Animals were given at least 1 week recovery time before being used for any behavioral experiments. For intracannulae injections, animals were anesthesized with isofluorane and 1 μl (5 μg) of JQ1 per side was injected at a rate of 0.3 μl min^−1^.

### Tissue dissection, RNA extraction and qRT-PCR

Animals were killed via cervical dislocation, the brain quickly removed on ice and hippocampal subregions dissected in ice-cold PBS under a dissection microscope. For the RNA-Seq experiment, animals were killed 24 h after the probe test after 13 (WT-JQ1) and 10 (APP-JQ1) injections. Tissue was snap-frozen in liquid nitrogen and stored at −80 °C until processing. RNA extraction was done using Tri reagent (Sigma) following manufacturer’s instructions. DNaseI treatment was performed prior to RNA-Seq to ensure no carry-over of genomic DNA. DNaseI-digested RNA was purified using Phenol:Chloroform according to the standard protocols. RNA was checked for concentration and purity in the Bioanalyzer (Agilent, Waldbronn, Germany). cDNA synthesis was done using Roche’s ‘First strand cDNA synthesis kit’ according to the manufacturer’s instructions. At least 1 ng of cDNA was used per qPCR reaction. Every reaction was run at least in duplicate to minimize the effect of pipetting variability. Data were analyzed with the DDCt method. A primer list is provided in Supplementary Table S6.

### RNA-Seq data analysis and reanalysis of publicly available data sets

RNA-Seq was carried out in a HiSeq2000 according to the manufacturer’s instructions. Reads were aligned to mm10 using STAR^25^ with the following parameters —outFilterMismatchNmax 2 —outSAMstrandField intronMotif —sjdbOverhang 49 and counts were generated using htseq.^26^ Downstream analysis was carried out using cufflinks/cuffdiff^27^ and deseq2.^28^ Genes were considered significant with an adjusted *P*-value under 0.05. Gene set overlaps were calculated using Venny.^29^ Functional enrichment analysis was performed using Webgestalt 2.0^30^ and gene network visualization was carried out using Cytoscape 3.0’s plugin ClueGO.^31^ Pathways were considered significant at an adjusted *P*-value cutoff of 0.1. RNA-Seq data have been deposited with GEO’s accession number GSE93796. For data meta-analysis, data from Shahbazi *et al.* (PMID 26733615) were analyzed with GEO2R and genes differentially affected by JQ1 were used as input for ClueGO.

### Hippocampal cultures and JQ1 treatment

Hippocampal cultures were prepared from E17 embryos from CD1 pregnant females. Females were quickly killed by cervical dislocation, embryos decapitated and hippocampi dissected on ice. Tissue was digested with 0.25% trypsin for 15 min at 37 °C, washed 3 × with DMEM containing 10% FBS and homogenized with 10–12 strokes of a P1000. Cells were then counted and plated in Neurobasal containing 20% B27 and 1% penicillin/streptomycin at 130 000 cells per well on 24-well plates (for RNA) or 700 000 cells per well (for protein) in 6-well plates. Cells were fed by exchanging 30% of the medium with fresh neurobasal medium every 2–3 days. On DIV 8, cells were treated with DMSO (vehicle) or JQ1 (ApexBio) at 250 nM. Cells were collected for RNA/protein extraction after 24 h or 30 min as indicated. RNA was extracted with Tri reagent as described above.

### Chromatin-bound protein fractionation and western blot

Cultures were prepared as described above and cells collected in PBS, spun and flash-frozen in liquid nitrogen at 24 h and 30 min post treatment with JQ1. The chromatin-bound fraction was isolated using ThermoFisher’s kit #78840 following manufacturer’s instructions. The resulting protein was loaded onto a 4–20% gradient precast gel (Bio-Rad, Munich, Germany) and run for 2 h at 80 V. Protein was transferred onto nitrocellulose membranes using Bio-Rad’s semi-dry transfer system. Antibody incubation was done overnight at 4 °C in TBST containing 0.5% BSA with gentle agitation. Antibodies used were as follows: BRD4 (Abcam 128874 1:200, Cambridge, UK) and H3 (Abcam 1791 1:1000). Blots were scanned in a Licor system.

### Slice preparation and electrophysiological measurements

Acute hippocampal slices were prepared as previously described^32^ from 10- to 12-week-old C57B/6j or 8- to 10-month-old APP/PS1–21 mice (see above). Animals were anesthetized with isoflurane and decapitated. Brain was removed from the skull and hippocampus was dissected. Transversal hippocampal slices (400 μm thick) were obtained using a tissue chopper (Stoelting, Wood Dale, IL, USA). Slices were collected in ice-cold artificial cerebrospinal fluid (124 mM NaCl, 4.9 mM KCl, 1.2 mM KH_2_PO_4_, 2.0 mM MgSO_4_, 2.0 mM CaCl_2_, 24.6 mM NaHCO_3_, 10.0 mM d-glucose; saturated with 95% O_2_ and 5% CO_2_; pH 7.4 and 305 mOsm). Slices were incubated in an interface chamber at 32 °C and high oxygen tension was maintained by bubbling with 95% O_2_ and 5% CO_2_ (30 l h^−1^). Slices were allowed to recover for 3 h after preparation. Then monopolar platinum–iridium electrodes (13303, MicroProbes, Gaithersburg, MD, USA), used for both recording and stimulating, were positioned in the CA1 region. The field excitatory postsynaptic potential slope was recorded with a Model 1700 differential AC amplifier (A-M Systems, Sequim, WA, USA) and Power 1401 analog-to-digital converter (Cambridge Electronic Design, Camebridge, UK), and monitored on-line with custom-made software, PWIN (IFN Magdeburg). The test stimulation strength was determined for each input as the current needed to elicit a field excitatory postsynaptic potential of 40% maximal slope. Baseline recording began at least 3.30 h after slice preparation, using test stimuli consisting of four biphasic constant-current pulses (0.2 Hz,0.1 ms per polarity, averaged) per time point, every 5 min for a minimum of 30 min. For WT slices, LTP was induced with a strong tetanization protocol consisting of three stimulus trains (100 biphasic constant-current pulses per train at 100 Hz and 0.2 ms per polarity, inter-train interval 10 min). For APP slices, the inter-train interval was 20 s. Test stimuli were delivered 1, 3, 5, 11, 15, 21, 25 and 30 min after the first tetanization train and then every 5 min for up to 2 h. The drug (JQ1, 250 nM) was applied from 30 min prior to stimulation and during the whole recording time.

### Statistical analysis

All statistical analyses were performed within GraphPad Prism. Bars represent mean±s.e.m. Statistical tests and exact *n* numbers are indicated in the figure legends.

## Results

Before starting with *in vivo* injections, we validated that in our hands JQ1 was able to displace BRD4 from the chromatin-bound fraction at 30 min and 24 h post treatment in culture (Supplementary Figure S1). Once we validated the effect of JQ1, we tested the effect of BET inhibitors on cognition. To this end, we injected young (3-month-old) wild-type (WT) mice intraperitoneally (i.p.) with vehicle or JQ1, a potent and widely used BET inhibitor that shows therapeutic effects in experimental models for various cancers.^14, 33^

**Figure 1:**
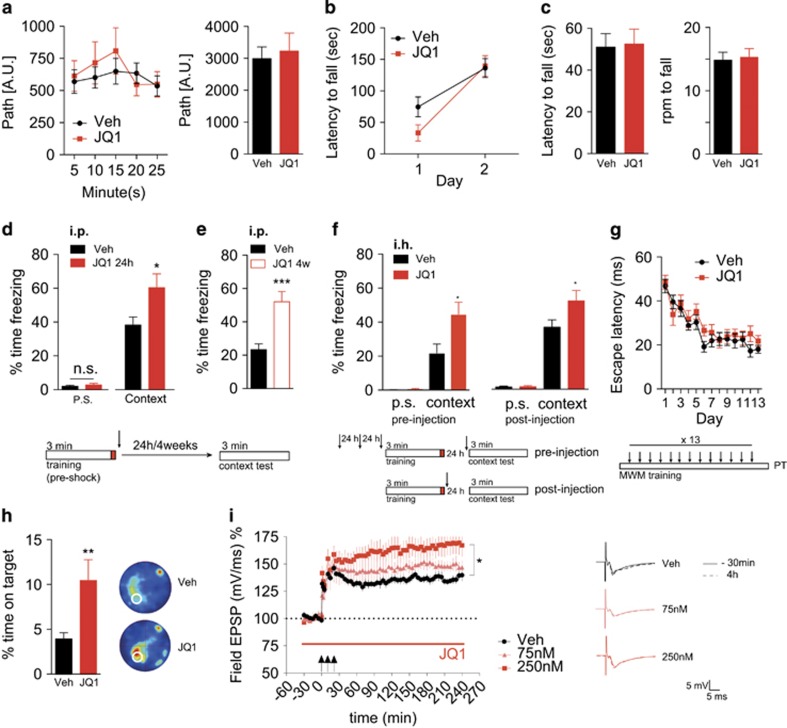
JQ1 improves cognitive performance in the fear-conditioning and water maze test without affecting basal motor and exploratory behavior. (**a**) Left panel: path per minute in the open field was not changed in JQ1-injected animals. *n*=10 (vehicle); 9 (JQ1). Right panel: total path in the open field was not changed in JQ1-injected animals. *n*=10 (vehicle); 9 (JQ1). (**b**) The latency to fall during the training phase of the rotarod test did not show significant differences between vehicle- and JQ1-injected animals. *n*=14 (vehicle); 14 (JQ1). (**c**) Left panel: latency to fall from the rod in the rotarod test was not different in JQ1-treated animals. *n*=14 (vehicle); 14 (JQ1). Right panel: the speed at which animals fell in the rotarod test was not different for JQ1-treated animals. *n*=14 (vehicle); 14 (JQ1). (**d**) A single JQ1 i.p. injection after training the contextual fear-conditioning paradigm led to increased freezing levels in JQ1-treated animals. *P<0.05, two-tailed Student’s *t*-test. *n*=8 (vehicle); 10 (JQ1). (**e**) Freezing levels are elevated in JQ1-treated animals when they were tested 4 weeks after training. ***P<0.001, two-tailed Student’s *t*-test. *n*=15 (vehicle); 15 (JQ1). (**f**) Left panel: JQ1 injections into the hippocampus (i.h.) before the training led to a significant increase in freezing levels in wild-type animals. *P<0.05, two-tailed Student’s *t*-test. *n*=9 (vehicle); 10 (JQ1); right panel: a single intrahippocampal JQ1 injection immediately after training in the fear-conditioning paradigm caused a borderline significant increase in freezing levels at an alpha level of 0.1. *P*-value from a two-tailed Student’s *t*-test. *n*=9 (vehicle); 8 (JQ1). (**g**) Escape latency of wild-type animals that received daily JQ1 injections after the training session in the MWM performed is similar to that of vehicle-injected animals. *n*=18 (vehicle); 16 (JQ1). (**h**) JQ1-treated animals spent significantly more time in the target area where the escape platform was during training, in the probe test. **P<0.01, two-tailed Student’s *t*-test. *n*=18 (vehicle); 16 (JQ1). Left panel shows heatmap occupancy plots representing the probe test for vehicle- and JQ1-injected animals. Brighter colors indicate higher occupancy. (**i**) Dose-dependent increase in LTP recorded from wild-type slices treated with vehicle or with increasing doses of JQ1 per bath application. *P<0.05, repeated measures ANOVA, F (1,12)=5.127; *n*=5 (vehicle); 10 (JQ1). Error bars indicate s.e.m. EPSP, excitatory postsynaptic potential; MWM, Morris Water Maze.

Daily JQ1 injections at a 50 mg kg^−1^ dose for 1 week prior to behavioral testing did not affect exploratory behavior in the open field test (Figure 1a). We also tested motor behavior in the rotarod paradigm. JQ1-treated animals did not significantly differ from the vehicle group during training (Figure 1b) or testing (Figure 1c). These data show that daily JQ1 administration did not interfere with explorative behavior and motor function. To test whether JQ1 affects memory consolidation, mice received a single i.p. injection of JQ1 immediately after contextual fear-conditioning training (Figure 1d), thereby ensuring that the injection does not interfere with the training procedure. When tested 24 h later, JQ1-treated animals displayed higher freezing behavior, indicative of enhanced memory consolidation (Figure 1d). In another group of mice, we also tested remote memory. To this end, we employed the same experimental setting but tested freezing behavior not 24 h later but only 4 weeks after the training. Of note, freezing behavior was significantly increased in JQ1-treated mice (Figure 1e), suggesting that JQ1 treatment has a lasting effect on consolidated memories. Although JQ1 and other BET inhibitors exhibit good blood–brain–barrier permeability,^34^ we wanted to specifically test whether the memory enhancing effects of JQ1 are mediated via its action in the brain. To this end, we implanted microcannulae into the dorsal hippocampus of mice and injected vehicle or JQ1 either before or immediately after the training (Figure 1f). In both experiments, JQ1-injected mice displayed enhanced freezing behavior when tested 24 h later (Figure 1f), suggesting that JQ1 improves memory consolidation via processes that involve hippocampal function. Therefore we also measured hippocampus-dependent spatial memory in the Morris Water Maze paradigm. Mice received daily i.p. injections of JQ1 (50 mg kg^−1^) starting on the first training day immediately after the training session (Figure 1g). While JQ1- and vehicle-treated animals showed similar escape latencies (Figure 1g), when subjected to the probe test, mice that had received JQ1 exhibited a significantly increased platform occupancy (Figure 1h). These results indicate that JQ1 can enhance at least two types of long-term memory without affecting basal exploratory or motor behavior. Next, we decided to examine synaptic plasticity in JQ1-treated acute hippocampal slices. We observed a dose-dependent increase in LTP in WT slices treated with JQ1 (Figure 1i). In conclusion, to the best of our knowledge, our data represent the first report that a BET inhibitor improves memory function and synaptic plasticity.

**Figure 2:**
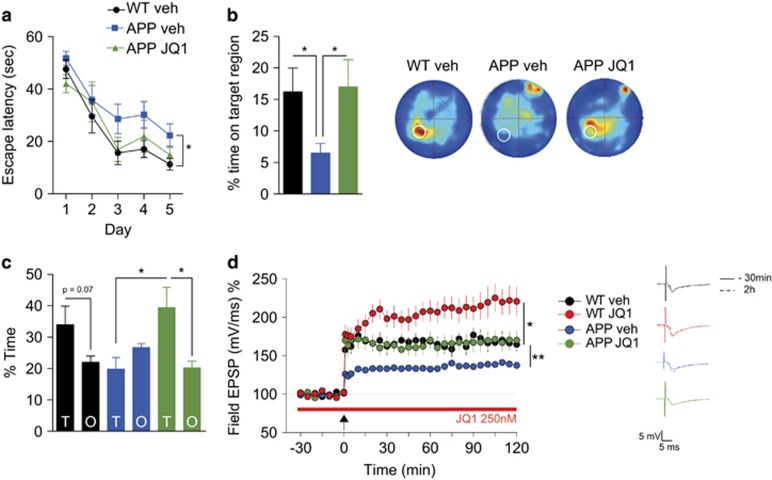
**J**Q1 rescues cognitive and electrophysiological deficits in 8-month-old APP/PS1–21 animals. (**a**) Escape latency curve in the MWM for wild-type vehicle, APP vehicle and APP-JQ1 animals. *P<0.05, repeated measures ANOVA wild-type vehicle vs APP vehicle; F (1, 16)=5.555; *n*=9 (wild-type vehicle); 9 (APP vehicle); 7 (APP JQ1). (**b**) (left panel) The percentage of time spent in the target area of the platform is significantly decreased in APP vs wild-type animals and rescued in APP-JQ1 animals. *P<0.05, two-tailed Student’s *t*-test. *n*=9 (wild-type vehicle); 9 (APP vehicle); 7 (APP JQ1). Right panel: heatmap occupancy plots representing the probe test for wild-type and APP animals. Brighter colors indicate higher occupancy. (**c**) The percentage of time spent in the target quadrant was significantly increased in APP vehicle vs APP-JQ1 animals. Wild-type animals also showed borderline significant preference for the target over the rest of the pool at an alpha level of 0.1, whereas APP-vehicle animals did not. APP-JQ1 animals showed a clear preference for the target quadrant. *P<0.05, two-tailed Student’s *t*-test. *n*=9 (wild-type vehicle); 9 (APP vehicle); 7 (APP JQ1). (**d**) LTP was enhanced in wild-type slices, reduced in APP-vehicle animals and rescued in APP animals by bath application of JQ1. *P<0.05, repeated measures ANOVA, wild-type vehicle vs wild-type JQ1. F (1, 13)=5.789. *n*=7 slices, 5 animals (wild-type vehicle); *n*=9 slices, 5 animals. **P<0.01, repeated measures ANOVA, wild-type vehicle vs APP vehicle. F (1, 9)=12.76. *n*=7 slices, 5 animals (wild-type vehicle); *n*=5 slices, 3 animals (APP vehicle). APP vehicle vs APP JQ1 comparison: *P*<0.05, F (1, 8)=8.902; *n*=5 slices, 3 animals (APP vehicle); *n*=5 slices, 3 animals (APP JQ1). Error bars indicate s.e.m. EPSP, excitatory postsynaptic potential; MWM, Morris Water Maze.

Encouraged by these data, we decided to test whether JQ1 would ameliorate memory defects in an animal model for amyloid pathology. To this end, we employed the APP/PS1–21 line (from here on, termed ‘APP animals’) at an age where pathology is already fully present (8 months of age). JQ1 injection did not cause any motor deficits in APP animals (Supplementary Figure S2). As expected, APP animals injected i.p. with vehicle solution performed poorly in the MWM test both during the acquisition phase (Figure 2a) and in the probe test (Figure 2b). Strikingly, JQ1 injections significantly improved the performance of APP mice during the training (Figure 2a) and during the probe test, as measured via the time spent on the target region (Figure 2b) and the time spent in the target quadrant (Figure 2c). In an additional group of mice, we also measured memory function in the pavlovian fear-conditioning paradigm. Using one-way ANOVA, we failed to detect a significant effect among groups, indicating that fear memories were not significantly impaired in APP mice at the tested age. A non-significant trend for memory impairment and JQ1-mediated rescue was however obvious (Supplementary Figure S3). In an additional group of mice, we also measured hippocampal LTP. While LTP was severely impaired in vehicle-treated APP mice compared to WT control, LTP measured in slices from APP animals treated with JQ1 was restored to the level of vehicle-treated WT control mice (Figure 2d). LTP in WT control mice treated with JQ1 was significantly increased, confirming our data obtained in 3-month-old mice (Figure 2d; see also Figure 1i). These data suggest that JQ1 can enhance memory and hippocampal plasticity in WT mice and restore it in a mouse model for neurodegeneration.

**Figure 3:**
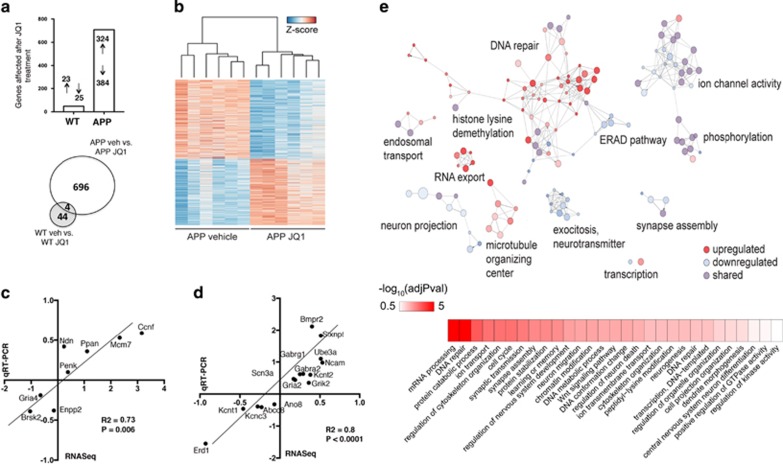
JQ1-induced gene expression in WT and APP mice. (**a**) Upper panel: number of up- and downregulated genes affected by JQ1 treatment in WT and APP mice. Lower panel: Venn diagram showing limited overlap of genes affected in WT and in APP mice. (**b**) Heatmap of differentially expressed genes in APP-vehicle vs APP-JQ1 animals showed two main clusters of up- and downregulated genes. (**c**) qPCR was performed for selected genes affected by JQ1 treatment in WT mice. Correlation analysis shows that qPCR data were able to confirm RNA-seq findings. (**d**) qRT PCR was performed for selected genes affected by JQ1 treatment in APP mice. Correlation analysis shows that qPCR data were able to confirm RNA-seq findings. (**e**) Network of functional categories represented by JQ1-associated changes in APP animals based on the Biological Process ontology. The size of the nodes correlates inversely with statistical significance. Categories of upregulated genes are red, downregulated genes are blue and those where there are both up- and downregulation are purple.

Previous data reported conflicting results of JQ1 treatment on immediate early gene (IEG) expression.^23, 35^ Thus, we examined a panel of IEGs after acute (30 min) and long-term (24 h) JQ1 treatment in hippocampal neurons. JQ1 had distinct effects on IEG expression both at 30 min and 24 h (Supplementary Figure S4). Of note, the 30 min treatment protocol increased the expression of relevant IEGs that are linked with the LTP, including Egr1, Egr2 and Junb while cFOS levels were only increased 24 h after JQ1 treatment (Supplementary Figure S4). Other IEG such as Arc and Nr4a2 were however downregulated, indicating that JQ1 has a complex effect on gene expression. In order to better understand the molecular basis for memory enhancement in WT and in APP animals, we carried out an RNA-Seq experiment from the CA1 region of the hippocampus. In WT mice, 48 genes were differentially expressed (25 decreased, 23 increased) after JQ1 treatment (Figure 3a, Supplementary Table 1), while in JQ1-treated APP mice we observed 708 differentially expressed genes (Figures 3a and b; 384 decreased, 324 increased, Supplementary Table 2). Differential expression of selected target genes was confirmed via qPCR in WT (Figure 3c) and in APP mice (Figure 3d). Of note, JQ1 treatment had no effect on the expression of the APP and PS1 transgenes in APP mice (Supplementary Table 3).

**Figure 4:**
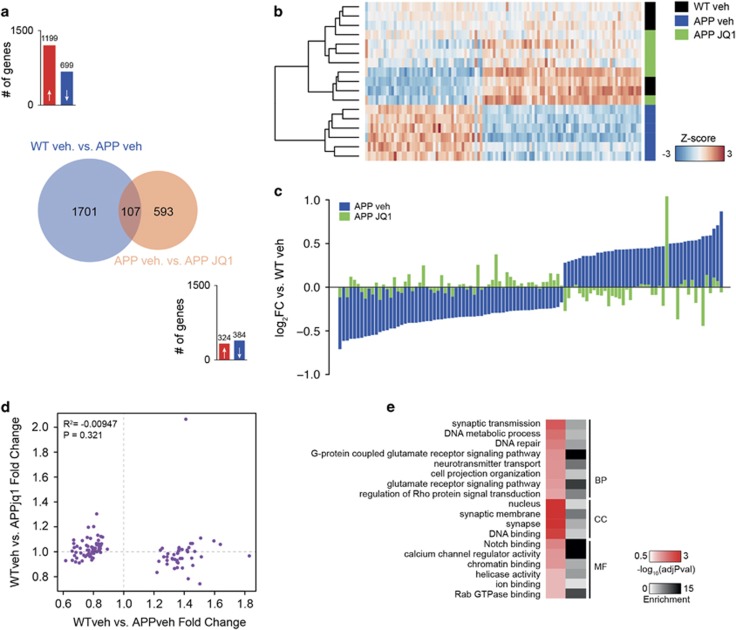
JQ1 restores physiological expression of a subgroup of amyloid-associated genes. (**a**) Venn diagram showing the overlap between purely APP-associated changes in JQ1-associated changes in APP animals. The graphs at the top and bottom of the Venn diagram show the number of up- and downregulated genes for the two conditions. (**b**) Heatmap for the 107 genes in the overlap between the APP and the APP-JQ1 signature shows partial restoration of physiological expression levels compared to the APP-vehicle signature. (**c**) Bar graph illustrating that for a subset of 107 genes, JQ1 was able to almost fully restore physiological gene expression. The figure shows the fold changes in APP/PS1–21 vehicle animals (vs wild-type animals) in blue and the fold changes in APP/PS1–21 vehicle animals (vs APP/PS1–21 JQ1 animals) in green. (**d**) Salient categories represented in the 107 genes in the overlap. Both enrichment and significance are represented. BP, biological process; CC, cellular component; MF, molecular function.

Pathway analysis revealed that genes affected by JQ1 treatment in APP mice represent networks related to ion channel activity, DNA repair, RNA localization mechanisms, transcription and so on (Figure 3e). Although only a small and distinct set of genes was regulated in JQ1-treated WT mice (Figure 3a), functional analysis revealed that these genes also represent a pathway linked to ion channel activity (adjusted *P*-value=0.043), which is in line with the data obtained in APP mice. The fact that JQ1 regulates a large gene-expression network in APP mice but has a comparatively mild effect in WT mice might be linked to the fact that chromatin plasticity is deregulated in APP mice,^36^ thus allowing drugs that act on the epigenome to exert a more pronounced action. Similar data have been observed in case of HDAC inhibitors that were shown to reinstate transcriptional homeostasis in models for neurodegeneration.^37^ Thus, we also tested to what extent JQ1 treatment may restore physiological gene expression in APP mice. To this end, we contrasted the gene sets detected when comparing WT vehicle to APP vehicle (1808 genes) and APP-vehicle to APP-JQ1-treated mice (700 genes). We observed 107 commonly regulated genes (Supplementary Table S4 and Figure 4a). When we subjected these 107 genes to hierarchical clustering, we observed a significant restoration of physiological expression values after JQ1 treatment (Figures 4b and c). In fact, JQ1-treated APP mice were more closely clustered with vehicle-treated WT mice, then with vehicle-treated APP mice (Figure 4b). Interestingly, functional enrichment analysis for this set of genes revealed pathways linked to neurotransmitter transport, glutamate response, synaptic transmission and DNA repair terms (Figure 4d), which is in line with the restoration of hippocampal LTP in JQ1-treated APP mice. As an additional validation, we also re-analyzed a published paper describing the effect of JQ1 on neuroblastoma SK-N-BE cells.^38^ As expected, we found that the overall effects are different, nevertheless in line with our observations, JQ1 had a consistent effect on neuronal development, axonal growth, membrane potential and ion channel transport, as well as DNA damage response (Supplementary Figure S5).

## Discussion

In this study, we show that systemic or intrahippocampal injection of JQ1 improves memory performance in WT mice. Moreover JQ1 enhances hippocampal LTP in a dose-dependent manner. JQ1 treatment also reinstates memory function and LTP in APP mice suggesting that small-molecule inhibitors of BET/BRD reader proteins could be a novel therapeutic strategy to treat AD. Our findings that JQ1 improves memory function and synaptic plasticity are in conflict with another study. Thus, Korb *et al.*^39^ failed to observe any effect on JQ1 treatment on contextual or cued fear-conditioning learning but suggest that JQ1 affects context generalization. The discrepancy to our findings might be explained by the employed fear-conditioning paradigm. Memory strength in the fear-conditioning paradigm is correlated to the intensity of the foot shook applied during training.^40^ Generally, milder foot shock protocols are employed if an experiment should enable not only the detection of memory deficits but also the potential detection of memory enhancement. Strong training protocols exclude the detection of memory enhancement due to ceiling effects. The fact that Korb *et al.* subjected mice to three electric foot shocks of 0.7 mA per 2 s, whereas in our case mice received only a single foot shock of 0.5 mA could thus explain the inconsistent findings. Korb *et al.*^23^ also present data that JQ1 administration impairs novel object recognition (NOR) learning in mice. This assay does not critically depend on hippocampal function^41^ and it is thus possible that systemic administration of JQ1 improves hippocampus-dependent memory function and synaptic plasticity, while it impairs other forms of cortical plasticity, an issue that should be addressed in future research. The interpretation that JQ1 might exert brain region-specific effects is supported by the finding that in our hands JQ1 treatment neither impaired nor enhanced NOR learning (Supplementary Figure S6). In addition, one has to take into account, that the NOR test is not the most suitable assay to detect memory enhancement in cognitively normal WT mice. Another study employed the BET/BRD inhibitor I-BET858 and observed no behavioral alterations when the drug was applied to adult mice, while administration of I-BET858 to juvenile animals led to autism-like phenotypes^35^ suggesting that BRD proteins play an important role in post-natal development.

Although obviously more research is needed to understand how the modulation of BET/BRD reader proteins impacts on memory function, we like to stress the fact that our data showing JQ-1-induced memory improvement in WT mice is supported by LTP measurements. Although behavioral testing of mice is known to be affected by many confounding factors including housing conditions or even the gender of the experimenter,^42^ which could also contribute to the apparently conflicting results across studies, the analysis of LTP in hippocampal slices is less prone to such effects and is considered a molecular correlate for memory formation. Of note, LTP has not been tested so far in the context of BET/BRD inhibitors and we show here that JQ1 clearly enhances LTP in a dose-dependent manner. Moreover, no BET/BRD inhibitors have been tested for their action in the context of neurodegenerative diseases. Thus, in addition to the data obtained in WT mice, we show for the first time that JQ1 improves memory function and hippocampal LTP in APP mice.

To our knowledge, our study is not only the first to report beneficial effects of JQ1 on memory and hippocampal LTP, but also the first to describe the effects of JQ1 on gene expression in the adult hippocampus *in vivo*. It is therefore interesting that one of the major gene-expression networks affected by JQ1 is related to ion channel activity, a finding that is in line with data obtained from *in vitro* experiments.^22, 39^ Another interesting observation is the fact that the same dose of JQ1 elicits a much greater change in hippocampal in gene expression in APP mice when compared to WT mice. This finding suggests that JQ1 might elicit different responses depending on the cellular milieu and chromatin state. It is therefore tempting to speculate that in a healthy physiological setting, drugs such as JQ1 exhibit only a moderate effect on gene expression. However, in a pathological setting such as AD pathogenesis, where chromatin dynamics and transcriptional plasticity are disrupted, drugs like JQ1 can exert a more pronounced effect and potentially help to reinstate transcriptional homeostasis. This interpretation is in line with the gene-expression changes observed in the hippocampus from JQ1-treated WT mice vs JQ1-treated APP mice and suggests that JQ1 could be a suitable strategy to treat AD. Further support for this interpretation stems from recent data showing that JQ1 mediates anti-inflammatory actions in several disease models.^43, 44, 45^ Of note, JQ1 was also found to decrease the inflammation and Tau phosphorylation in an 3 × TG mice, another animal model for AD.^24^ It is in this context important to mention that JQ1 may affect other processes than chromatin plasticity and gene expression. The fact that JQ1 has, for example, very distinct effects on IEG expression when applied to hippocampal neurons such as increasing *cFos* but decreasing *Arc* expression supports the view that gene expression may not fully explain the memory enhancing effect of JQ1. Moreover, robust changes in the expression of IEG were observed when JQ1 was administered to hippocampal neurons while data from the *in vivo* experiments was less conclusive suggesting that the cellular context and duration of JQ1 treatment distinctly dictates gene-expression changes. This may also—at least in part—explain the discrepancy to two previous studies that either observed no change or decreased IEG expression after JQ1 treatment in different experimental settings.^23, 35^ Although there is no evidence available yet, BET/BRD proteins may also recognize other proteins than acetylated histones. This is of particular interest, as non-histone protein acetylation has been implicated with numerous cellular processes.^46^ Thus, it will be important for future studies to test whether JQ1 would elicit proteome or acetylome changes. This may also help to better understand our observation that JQ1 improves memory function and LTP in wild-type mice and in a mouse model for AD. It is possible that these effects are mediated via different mechanisms. The observation that JQ1-induced changes in gene expression differ substantially when comparing wild-type and APP mice supports this view.

In summary, BET/BRD inhibitors are currently discussed as promising targets in cancer research and several clinical trials have been initiated (www.clincaltrials.gov). It is thus of utmost importance to understand whether systemic administration of drugs such as JQ1 would affect brain function. To learn more about BET/BRD inhibitors in translational neuroscience is furthermore important, taken into account that drugs which inhibit a related class of proteins, namely HDAC inhibitor, are already used in cancer treatment and also provide suitable drug targets to treat neurodegenerative diseases.^3^ Data on BET/BRD inhibitors and memory function are however rare and conflicting. Here we provide solid evidence that the BET/BRD inhibitor JQ1 improves hippocampal memory function and LTP in WT mice and in a mouse model for AD, which is linked to a substantial change in hippocampal gene expression. Our results are encouraging for the development of dementia therapies or to treat age-associated memory decline and suggest that BET inhibitors should be further tested in other animal models of cognitive impairment.
