# Supplementary material for: The BET/BRD inhibitor JQ1 improves brain plasticity in WT and APP mice
Source: Transl Psychiatry. 2017 Sep 26;7(9):e1239–. doi: 10.1038/tp.2017.202 (PMC5639246; doi:10.1038/tp.2017.202)
Supplement: Supplementary Tables [file tp2017202x1.pdf]

Table S1. Genes differentially expressed in JQ1 treated wild type mice

Gene Symbol	log2FoldChange	adjusted p-value
Mir181a-1,mmu-mir-181b-1	3,49	2,43E-02
Apba3	0,47	2,43E-02
Trpm2	0,97	2,43E-02
Dvl2	-1,13	2,43E-02
1810032O08Rik	1,17	2,43E-02
Slc25a47	1,91	2,43E-02
Ep300	3,38	2,43E-02
Mir30b,RP23-61D6.4	-2,70	2,43E-02
Gm20732,Mir30d	-5,19	2,43E-02
Rtn4r	0,28	2,43E-02
Rtp1	1,45	2,43E-02
Vgll3	-0,61	2,43E-02
2810055G20Rik,Mir99a,Mirlet7c-1	2,38	2,43E-02
Mir155,mmu-mir-155	-7,86	2,43E-02
Ccnf	3,16	2,43E-02
1110038B12Rik,Gm24101,Gm25744,Snord52	-2,24	2,43E-02
Capn11	1,08	2,43E-02
AA467197	4,49	2,43E-02
Gm10800	-0,69	2,43E-02
Bcas1	-0,28	2,43E-02
Cbln4	0,43	2,43E-02
Focad	-0,87	2,43E-02
Penk	0,39	2,43E-02
Ubap2	-1,25	2,43E-02
Rcan3	-0,67	2,43E-02
Mcm7	2,33	2,43E-02
Gm3294,Mir129-1	-1,93	2,43E-02
Pdia4	-0,52	2,43E-02
Brsk2	-1,04	2,43E-02
Kctd15	0,50	2,43E-02
BC017158	1,76	2,43E-02
Mir24-2,RP24-64D24.4	-1,32	2,43E-02
Ppan	1,11	2,43E-02
Lars2	0,37	2,43E-02
Gria4	-0,65	2,43E-02
Glce	-1,21	2,43E-02
AC163666.1,Gm23017	-5,68	2,43E-02
Pcsk1n	0,37	2,43E-02
Tlr13	-1,85	2,43E-02
Cstf2	-0,58	2,43E-02
-	0,41	2,43E-02
Slc47a1	-0,65	4,15E-02
Hist1h1c	-0,33	4,15E-02

Trpm3	-0,27	4,15E-02
Opalin	-0,30	4,15E-02
C1qtnf4	0,38	4,15E-02
Gm10801	-0,93	4,15E-02
Ndn	0,24	4,15E-02

Table S2: Genes differentially expressed in JQ1 treated wild type mice. Comparision of RNA-seq and qPCR results

Gene Symbol	RNASeq log2FC	qRT-PCR log2FC
Ccnf	3,16	0,59
Gria4	-0,65	-0,19
Mcm7	2,33	0,53
Ndn	0,24	0,42
Ppan	1,11	0,36
Brsk2	-1,04	-0,39
Enpp2	-0,15	-0,38
Penk	0,39	0,10

Table S3: RNA-seq reveals genes differentially expressed when comparing vehicle and JQ-1 treated APP mice

Gene Symbol	log2FoldChange	adjusted p-value
Gucy1a2	2,46	2,4E-27
Lnpep	2,58	1,7E-26
Xkr4	2,11	1,2E-21
Ptpn4	2,03	4,4E-21
BC005561	2,13	2,8E-13
Vps13a	0,81	1,4E-12
Fzd3	1,82	1,8E-11
Ube3a	1,10	1,2E-10
Sfpq	0,59	1,4E-10
Stxbp5l	1,83	3,0E-10
Slc16a7	1,09	2,5E-08
Mmp16	0,84	2,3E-07
Fam135b	1,79	1,2E-06
Clock	0,65	3,2E-06
Atm	0,73	3,6E-06
Smc2	0,68	4,0E-06
NA	-2,08	4,7E-06
Smg1	0,68	4,9E-06
Ccdc88a	0,59	6,1E-06
Usp9x	0,96	6,9E-06
4632427E13Rik	1,53	8,7E-06
Gabrb1	0,85	1,0E-05
Wnk3	0,61	1,0E-05
Arhgap5	0,86	1,1E-05
Lzts2	-0,45	1,1E-05
Bmpr2	2,12	1,1E-05
Scai	0,63	1,3E-05
4932438A13Rik	0,49	1,3E-05
Tnk2	-0,38	1,3E-05
Csmd3	0,67	1,3E-05
Adam11	-0,53	1,3E-05
Zfp628	-0,63	1,3E-05
Mtcp1	0,84	2,0E-05
Ttc14	0,39	2,0E-05
Ncor2	-0,67	2,0E-05
Bai1	-0,48	2,0E-05
Zfp871	1,85	2,4E-05
Dgkh	1,66	2,4E-05
A530054K11Rik	0,65	3,1E-05
Aatk	-0,37	3,1E-05
Map1s	-0,62	3,1E-05
Rev3l	0,53	4,6E-05

Thoc2	0,51	4,6E-05
Ankrd12	0,50	4,6E-05
Pdlim7	-0,58	4,6E-05
D10Bwg1379e	1,25	5,1E-05
B4galnt4	-0,38	5,2E-05
Onecut2	0,85	5,8E-05
Col19a1	1,03	6,5E-05
Atp13a2	-0,45	6,5E-05
Phip	0,59	6,9E-05
Lpcat4	-0,45	7,5E-05
Azi1	-0,54	8,2E-05
Pcsk1n	-0,86	8,6E-05
Pcdhb16	0,82	9,4E-05
Usp34	0,56	9,9E-05
Lrfn4	-0,57	1,1E-04
Atrx	0,53	1,2E-04
Tia1	0,32	1,2E-04
Dmd	0,47	1,3E-04
Zfp865	-0,53	1,3E-04
Zfp219	-0,54	1,3E-04
Nckap5l	-0,59	1,3E-04
Nrxn2	-0,39	1,5E-04
Rictor	0,49	1,5E-04
Ints1	-0,47	1,5E-04
Zfp120	0,60	1,8E-04
Ncln	-0,41	2,0E-04
Acsl3	0,33	2,4E-04
Lcor	1,78	2,6E-04
Zbtb37	1,27	2,6E-04
Ankrd13b	-0,42	2,7E-04
Nphp4	-0,53	2,7E-04
Cc2d1a	-0,35	3,1E-04
Cnot6	0,46	4,0E-04
NA	1,38	4,7E-04
Zfp945	0,53	4,7E-04
Rnf126	-0,50	5,1E-04
Mga	0,48	5,3E-04
Spns1	-0,41	5,3E-04
Jag2	-0,46	5,3E-04
Capn15	-0,49	5,3E-04
Pbrm1	0,45	5,4E-04
Grm4	-0,71	5,4E-04
BC016423	0,54	5,4E-04
Tmem240	-0,32	5,8E-04
Zhx1	0,39	6,5E-04
Pik3cd	-0,41	7,3E-04

Itgb8	0,60	7,3E-04
Dync2h1	0,49	7,3E-04
Pex6	-0,42	8,1E-04
Tmem201	-0,30	8,4E-04
Snrnp70	-0,38	8,5E-04
Prr7	-1,01	8,5E-04
Rif1	0,60	8,9E-04
Dos	-0,36	1,0E-03
Ncam2	0,98	1,3E-03
Abcb9	-0,41	1,3E-03
Lmtk3	-0,58	1,3E-03
Zfp397	0,42	1,3E-03
Mus81	-0,39	1,3E-03
Vars	-0,43	1,3E-03
Kctd17	-0,38	1,4E-03
Ggt7	-0,30	1,4E-03
Dmxl2	0,36	1,4E-03
Scrt1	-0,42	1,4E-03
Atr	0,46	1,4E-03
Gtpbp3	-0,41	1,5E-03
Prex2	0,66	1,5E-03
Cacna1h	-0,54	1,5E-03
Cntnap5b	1,34	1,6E-03
Lrp1b	0,65	1,6E-03
Dlk2	-0,61	1,6E-03
Col7a1	-0,93	1,7E-03
Fv1	0,82	1,8E-03
Vgf	-0,76	1,8E-03
Atg2a	-0,47	1,8E-03
Lonrf3	1,11	1,9E-03
Zfp26	0,60	1,9E-03
Slc15a2	0,52	1,9E-03
Ubald1	-0,46	1,9E-03
Ercc2	-0,52	1,9E-03
Ccdc85b	-0,69	1,9E-03
Mroh1	-0,48	2,0E-03
Zmiz2	-0,35	2,0E-03
Map2k2	-0,38	2,0E-03
Ccm2l	-0,80	2,0E-03
Frs3	-0,42	2,2E-03
Rap1gap	-0,35	2,3E-03
Nipbl	0,55	2,3E-03
Scaf1	-0,38	2,3E-03
4931428F04Rik	-0,45	2,4E-03
Dot1l	-0,50	2,4E-03
Sptbn4	-0,40	2,7E-03

Ppip5k2	0,32	2,7E-03
Klc2	-0,35	2,7E-03
Sirt6	-0,53	2,7E-03
Zfp292	0,54	2,8E-03
Mtdh	0,54	2,8E-03
Abca5	0,44	2,9E-03
Lrp3	-0,35	2,9E-03
Dmwd	-0,34	2,9E-03
Aars2	-0,38	2,9E-03
Hook1	0,38	2,9E-03
Fgf14	0,54	3,0E-03
Dazap1	-0,39	3,1E-03
Ssbp4	-0,53	3,1E-03
Tmem198	-0,46	3,2E-03
Ache	-0,59	3,2E-03
Amigo3	-0,79	3,4E-03
Ano8	-0,32	3,4E-03
Srsf10	0,36	3,5E-03
Tenm1	0,86	3,6E-03
Itgbl1	0,67	3,6E-03
Slc4a7	0,51	3,6E-03
Apba3	-0,50	3,6E-03
Rfx1	-0,53	3,6E-03
Abcc10	-0,60	3,6E-03
Zfp574	-0,42	3,6E-03
Shprh	0,41	3,7E-03
Kctd16	0,95	3,9E-03
Dlgap4	-0,34	4,0E-03
Pikfyve	0,95	4,0E-03
Trdmt1	0,57	4,0E-03
Usp15	0,39	4,0E-03
Vars2	-0,42	4,2E-03
Tjap1	-0,59	4,5E-03
Adck5	-0,41	4,7E-03
Inf2	-0,53	4,7E-03
Cep290	0,52	4,8E-03
Fbxl19	-0,34	4,8E-03
Xrn1	1,20	4,9E-03
Dek	0,36	4,9E-03
Lingo2	0,46	5,0E-03
Spag9	0,41	5,0E-03
Brwd3	0,62	5,1E-03
Rabgap1l	0,35	5,9E-03
Noc2l	-0,32	5,9E-03
Cadm4	-0,27	6,2E-03
Matk	-0,35	6,2E-03

Robo3	-0,71	6,4E-03
Eml5	0,39	6,4E-03
Zranb2	0,24	6,4E-03
Ttyh3	-0,32	6,6E-03
Speg	-0,42	6,6E-03
Fmnl1	-0,47	6,6E-03
Gabrg1	0,62	6,6E-03
Josd2	-0,56	6,6E-03
Slc9a7	1,11	6,7E-03
Ranbp2	0,51	6,7E-03
Aldh6a1	0,39	6,7E-03
Mib2	-0,34	6,9E-03
Pds5a	0,61	7,0E-03
Fubp1	0,25	7,2E-03
Dok6	1,42	7,3E-03
Atad2b	0,97	7,4E-03
Pcdhb19	0,67	7,5E-03
Cpsf6	0,30	7,5E-03
Ipo4	-0,33	7,5E-03
Slc7a6	-0,30	7,8E-03
Asic1	-0,36	7,8E-03
Emc10	-0,37	7,9E-03
Lingo1	-0,47	7,9E-03
Dmxl1	0,50	8,1E-03
Dhx36	0,32	8,1E-03
Dtx1	-0,30	8,1E-03
Eml2	-0,31	8,2E-03
Apbb3	-0,29	8,2E-03
Gga1	-0,33	8,2E-03
Agrn	-0,37	8,2E-03
Klhl7	0,35	8,2E-03
Nr1h2	-0,38	8,2E-03
Ckb	-0,37	8,3E-03
Wdr54	-0,35	8,4E-03
Elfn1	-0,43	8,4E-03
Ssr3	0,32	8,4E-03
Vezt	0,27	8,4E-03
Fastk	-0,34	8,4E-03
Kdm5a	0,39	8,4E-03
Jmjd1c	0,40	8,4E-03
Cic	-0,46	8,5E-03
Smg9	-0,44	8,5E-03
Rusc2	-0,35	8,5E-03
Stag2	0,39	8,6E-03
Wdfy1	0,37	8,6E-03
Ppard	-0,44	8,6E-03

Tpgs1	-0,53	8,6E-03
Shkbp1	-0,60	8,6E-03
Zfp329	0,38	8,7E-03
Hid1	-0,26	8,7E-03
Tmem63b	-0,29	8,7E-03
Dus3l	-0,32	8,7E-03
Zfr2	-0,45	8,8E-03
Fem1c	0,39	8,8E-03
Kcnh2	-0,41	8,8E-03
Ubr1	0,32	8,9E-03
Zfp191	0,35	9,0E-03
Gtpbp6	-0,39	9,0E-03
Prpf4b	0,31	9,1E-03
Stx4a	-0,28	9,1E-03
Ccdc9	-0,29	9,1E-03
Foxp4	-0,39	9,1E-03
Panx2	-0,39	9,1E-03
Zfp207	0,17	9,2E-03
Pik3c2a	0,42	9,2E-03
Slc2a6	-0,49	9,2E-03
Cdc37l1	0,28	9,2E-03
Fbrsl1	-0,41	9,2E-03
Crot	0,36	9,3E-03
Sf3b1	0,20	9,3E-03
Pagr1a	-0,34	9,5E-03
Mpp3	-0,26	9,6E-03
Vps13c	0,75	9,8E-03
Tmem167	0,38	9,8E-03
Wnk2	-0,39	9,9E-03
Psd3	0,42	9,9E-03
Whsc1	0,30	9,9E-03
Ppp1r12c	-0,25	9,9E-03
Numbl	-0,41	9,9E-03
Cipc	-0,23	9,9E-03
Myef2	0,31	1,0E-02
Yes1	0,46	1,0E-02
Gabra2	0,63	1,0E-02
Map7d1	-0,26	1,0E-02
Myh7b	-0,67	1,0E-02
Prrt3	-0,38	1,0E-02
Hsf1	-0,40	1,0E-02
Prss41	-0,78	1,0E-02
Mthfsd	-0,31	1,0E-02
Epha6	0,62	1,1E-02
Npdc1	-0,29	1,1E-02
Gigyf1	-0,44	1,1E-02

Zdhhc8	-0,40	1,1E-02
Bmpr1a	0,40	1,1E-02
Grin1	-0,35	1,1E-02
Dgki	0,61	1,1E-02
Capn7	0,26	1,1E-02
Mark4	-0,39	1,1E-02
Kif5b	0,58	1,1E-02
Rtn4rl2	-0,55	1,1E-02
Tle2	-0,56	1,1E-02
Fcrls	-1,48	1,1E-02
Ppp6r1	-0,25	1,1E-02
Dzip3	0,39	1,1E-02
Tmem120b	-0,48	1,1E-02
Pqlc3	-0,54	1,1E-02
Gpr3	-0,89	1,1E-02
Pcnxl3	-0,39	1,1E-02
Plch2	-0,41	1,1E-02
Foxo6	-0,54	1,2E-02
Pura	0,35	1,2E-02
Xiap	0,40	1,2E-02
Rhpn1	-0,45	1,2E-02
Hiatl1	0,33	1,2E-02
Gcn1l1	-0,33	1,2E-02
Tmc4	-0,89	1,2E-02
Grm3	0,60	1,2E-02
Zfp335	-0,42	1,2E-02
Arhgef1	-0,50	1,2E-02
Mar-07	0,35	1,3E-02
Iws1	0,43	1,3E-02
Lrrc7	0,54	1,3E-02
Nbeal1	0,50	1,3E-02
Phf20l1	0,26	1,3E-02
Rai1	-0,41	1,3E-02
Tnfrsf19	0,28	1,3E-02
Bai2	-0,35	1,3E-02
Sympk	-0,27	1,4E-02
Ccdc176	0,46	1,4E-02
Dagla	-0,43	1,4E-02
Tmem86b	-0,61	1,4E-02
Nup153	0,39	1,4E-02
Fam135a	0,34	1,4E-02
Plec	-0,54	1,4E-02
Lyst	0,55	1,4E-02
Gtpbp2	-0,32	1,4E-02
Tm6sf2	-0,67	1,4E-02
Sppl2b	-0,35	1,4E-02

Pkia	0,34	1,4E-02
Smc5	0,33	1,4E-02
Ndrg2	0,29	1,4E-02
Tgfb1i1	-0,60	1,4E-02
Fbrs	-0,35	1,5E-02
Cnot6l	0,35	1,5E-02
Dexi	-0,29	1,5E-02
Bag6	-0,29	1,5E-02
Top2b	0,36	1,5E-02
Slc9a5	-0,53	1,5E-02
N4bp2l2	0,42	1,5E-02
Mfsd7b	0,34	1,5E-02
Egr4	-0,93	1,5E-02
Hdgfrp2	-0,25	1,5E-02
Taf1c	-0,43	1,5E-02
Bmi1	0,32	1,6E-02
Chpf	-0,45	1,6E-02
Ap5z1	-0,47	1,6E-02
Nudc	-0,26	1,6E-02
Spred3	-0,34	1,6E-02
Dyrk1b	-0,58	1,6E-02
Akap9	0,38	1,6E-02
Ap2a1	-0,31	1,6E-02
Zbed6	1,64	1,6E-02
A330076H08Rik	0,47	1,6E-02
Capn1	-0,28	1,6E-02
Caskin1	-0,35	1,6E-02
Rbm26	0,29	1,6E-02
Taf1d	0,43	1,6E-02
Zfp518a	0,49	1,6E-02
Gpm6a	0,41	1,6E-02
Unc80	0,40	1,6E-02
Pxn	-0,40	1,6E-02
Fam179b	0,43	1,6E-02
Rora	0,43	1,6E-02
Bicd1	0,37	1,6E-02
Sh2d3c	-0,41	1,6E-02
Lrrc16b	-0,44	1,6E-02
Ppp2r5b	-0,23	1,7E-02
Fam172a	0,27	1,7E-02
Sst	-0,31	1,7E-02
Ndufs7	-0,36	1,7E-02
Grik5	-0,30	1,7E-02
Ska2	0,36	1,7E-02
Qpctl	-0,33	1,7E-02
Whrn	-0,50	1,7E-02

Tmem106b	0,37	1,8E-02
Rltpr	-0,36	1,8E-02
Slc27a4	-0,21	1,8E-02
Rps6ka5	0,36	1,8E-02
Inpp5j	-0,38	1,8E-02
NA	-0,51	1,8E-02
Slc36a1	-0,45	1,8E-02
Ahctf1	0,34	1,8E-02
Kcnc3	-0,39	1,8E-02
Alms1	0,76	1,8E-02
Cul4b	0,34	1,8E-02
Pank3	0,29	1,8E-02
Scn1b	-0,29	1,9E-02
Nckipsd	-0,26	1,9E-02
Mbd6	-0,49	1,9E-02
Ahdc1	-0,40	1,9E-02
Rps6kb2	-0,39	1,9E-02
C1qtnf4	-0,71	1,9E-02
Gstm6	0,61	1,9E-02
Yjefn3	-0,77	1,9E-02
Gas2l1	-0,41	1,9E-02
Rgs11	-0,44	1,9E-02
Pcm1	0,38	2,0E-02
Tyro3	-0,33	2,0E-02
NA	2,17	2,0E-02
Fgd4	0,46	2,0E-02
Wwp1	0,35	2,0E-02
Lypla2	-0,37	2,0E-02
Znhit2	-0,44	2,0E-02
Pcdh7	0,84	2,0E-02
Sh3bp1	-0,54	2,0E-02
Usp19	-0,26	2,0E-02
Med25	-0,42	2,0E-02
Tmem161a	-0,25	2,0E-02
Ccdc73	0,55	2,1E-02
Hip1r	-0,26	2,1E-02
Rasa1	0,38	2,1E-02
Pgp	-0,35	2,1E-02
Kbtbd8	0,47	2,1E-02
Klhl28	0,97	2,1E-02
Arid4b	0,35	2,1E-02
Scn3a	0,47	2,1E-02
4930485B16Rik	0,46	2,1E-02
Rock1	0,39	2,1E-02
Gpm6b	0,32	2,1E-02
Qtrt1	-0,31	2,1E-02

Taok1	0,40	2,1E-02
Pcdhb14	0,55	2,1E-02
Zfp53	0,68	2,2E-02
Rbl2	0,24	2,2E-02
Snx13	0,31	2,2E-02
Chst8	-0,49	2,2E-02
Mpp5	0,37	2,2E-02
Celsr3	-0,47	2,2E-02
Ptprz1	0,39	2,2E-02
Srek1ip1	0,26	2,3E-02
D14Abb1e	0,42	2,3E-02
Rb1cc1	0,36	2,3E-02
Tmem259	-0,29	2,3E-02
Zfc3h1	0,32	2,3E-02
Rnf207	-0,44	2,3E-02
Dvl1	-0,28	2,4E-02
Zfp758	0,72	2,4E-02
Trim41	-0,24	2,4E-02
Tspan17	-0,38	2,4E-02
Fsd1	-0,25	2,4E-02
Trpm7	0,24	2,4E-02
Ptprn	-0,35	2,4E-02
Ostm1	0,31	2,4E-02
Tfeb	-0,54	2,4E-02
Zranb1	0,32	2,4E-02
Ccnd3	-0,35	2,4E-02
Slc39a2	-0,51	2,4E-02
Phldb1	-0,48	2,4E-02
Tab3	0,37	2,4E-02
Kdm6a	0,33	2,4E-02
Ankrd13d	-0,28	2,4E-02
Ankrd9	-0,47	2,4E-02
Ranbp3	-0,19	2,4E-02
Erf	-0,34	2,4E-02
Trove2	0,42	2,4E-02
Map2	0,36	2,4E-02
Grik2	0,35	2,4E-02
Sfrs18	0,23	2,4E-02
Megf6	-1,28	2,4E-02
Xpo4	0,89	2,4E-02
Reps2	0,37	2,4E-02
Gpr162	-0,21	2,4E-02
Prrc2a	-0,35	2,4E-02
Ccdc34	0,35	2,4E-02
Plk3	-0,42	2,4E-02
NA	-0,56	2,4E-02

Tmem191c	-0,41	2,5E-02
Hps4	-0,44	2,5E-02
N4bp2	0,46	2,5E-02
Plekhh3	-0,40	2,5E-02
Gramd1a	-0,31	2,5E-02
Fam53b	-0,43	2,5E-02
Arsk	0,36	2,5E-02
Irf2	-0,30	2,5E-02
Wapal	0,37	2,5E-02
Vps13b	0,35	2,5E-02
Kctd4	0,38	2,5E-02
Folh1	0,82	2,6E-02
Appl1	0,33	2,6E-02
Esrra	-0,37	2,6E-02
Sp4	0,47	2,6E-02
Ppp1r37	-0,25	2,6E-02
Stk11ip	-0,29	2,6E-02
Trim46	-0,36	2,6E-02
Gltscr1	-0,43	2,6E-02
Fam57b	-0,43	2,6E-02
Erdr1	-1,55	2,6E-02
Rsbn1	0,60	2,6E-02
Hdac5	-0,31	2,6E-02
D930048N14Rik	-0,50	2,6E-02
Xist	0,50	2,6E-02
Megf8	-0,34	2,6E-02
Adk	0,34	2,6E-02
Ktn1	0,26	2,6E-02
Mfsd5	-0,27	2,6E-02
Ogt	0,22	2,7E-02
Tmem151a	-0,28	2,7E-02
Abca2	-0,33	2,7E-02
Atp13a1	-0,25	2,7E-02
Pip5k1c	-0,28	2,7E-02
Pprc1	-0,33	2,7E-02
Pik3r2	-0,27	2,7E-02
Abca7	-0,43	2,7E-02
Prkdc	0,35	2,7E-02
Pick1	-0,21	2,7E-02
Ccdc157	-0,29	2,7E-02
Rtn4r	-0,37	2,7E-02
Syvn1	-0,33	2,7E-02
Rps6ka4	-0,28	2,8E-02
Safb	-0,26	2,8E-02
Fuk	-0,35	2,8E-02
Ccdc92	-0,25	2,8E-02

Crocc	-0,56	2,8E-02
Eya3	-0,24	2,8E-02
Vcpip1	0,37	2,9E-02
Narg2	0,37	2,9E-02
Midn	-0,36	2,9E-02
Impdh1	-0,30	2,9E-02
Scrib	-0,40	2,9E-02
AW551984	0,56	2,9E-02
Ascc3	0,32	2,9E-02
Adam10	0,28	2,9E-02
Hgs	-0,25	2,9E-02
Rsf1	0,35	2,9E-02
Phkg2	-0,25	2,9E-02
Cntfr	-0,34	2,9E-02
Vps37b	-0,40	2,9E-02
NA	-0,40	2,9E-02
Ndnf	0,53	3,0E-02
Mybbp1a	-0,25	3,0E-02
Zfp385a	-0,42	3,0E-02
Chl1	0,41	3,0E-02
Slc35e4	-0,29	3,0E-02
Vcan	0,41	3,0E-02
Fam126b	0,50	3,0E-02
Arfgef1	0,33	3,0E-02
Tnpo1	0,28	3,0E-02
Msantd2	-0,31	3,0E-02
Arhgap33	-0,36	3,0E-02
2310003H01Rik	-0,44	3,0E-02
Phc2	-0,25	3,1E-02
Map3k2	0,35	3,1E-02
Chic1	0,32	3,1E-02
Atxn2l	-0,38	3,1E-02
Bnip2	0,28	3,1E-02
Zswim8	-0,29	3,1E-02
Arhgef17	-0,29	3,1E-02
Kcnj11	-0,44	3,1E-02
Dlgap3	-0,37	3,1E-02
Zdhhc21	0,30	3,2E-02
B3galt2	0,53	3,2E-02
Fam210a	0,30	3,2E-02
Ptprd	0,41	3,2E-02
Senp7	0,35	3,2E-02
Rapgef6	0,25	3,2E-02
Caprin1	0,23	3,2E-02
Dpysl4	-0,29	3,2E-02
Ptpn23	-0,39	3,2E-02

Fbxo31	-0,23	3,2E-02
Leng8	-0,40	3,2E-02
Ell	-0,27	3,2E-02
Safb2	-0,31	3,2E-02
Vps33b	-0,23	3,3E-02
Kcnt1	-0,46	3,3E-02
Erbb4	0,46	3,3E-02
Ralgapa1	0,28	3,3E-02
Map3k11	-0,31	3,3E-02
Rnf141	0,31	3,3E-02
Nim1k	0,28	3,3E-02
Pacsin1	-0,29	3,3E-02
Pip5kl1	-0,64	3,3E-02
Unc5a	-0,35	3,3E-02
Galt	-0,34	3,4E-02
Cp	0,71	3,4E-02
Spsb3	-0,45	3,4E-02
Fam171b	0,37	3,4E-02
Radil	-0,43	3,4E-02
Lrfn1	-0,39	3,4E-02
Wdr52	0,67	3,4E-02
Spag6	0,44	3,4E-02
Lrrc45	-0,36	3,4E-02
Pla2g3	-0,48	3,4E-02
Sec23a	0,29	3,5E-02
Sypl	0,26	3,5E-02
Ccne2	0,48	3,5E-02
Hkdc1	-0,67	3,5E-02
Cntnap5a	0,72	3,5E-02
Fam73b	-0,26	3,5E-02
Chpf2	-0,41	3,5E-02
Syne1	0,40	3,5E-02
Eci1	0,37	3,5E-02
Mon1a	-0,31	3,6E-02
Skiv2l	-0,32	3,6E-02
Recql5	-0,33	3,6E-02
Nfkbib	-0,33	3,7E-02
C77370	0,49	3,7E-02
Cpeb4	0,43	3,7E-02
Tmem184b	-0,27	3,7E-02
Cpne9	-0,92	3,8E-02
Scn9a	0,67	3,8E-02
Fpgt	0,37	3,8E-02
A230046K03Rik	0,32	3,8E-02
Btaf1	0,23	3,8E-02
Jund	-0,29	3,8E-02

Kifc3	-0,32	3,8E-02
Them6	-0,36	3,8E-02
Caskin2	-0,38	3,8E-02
Cherp	-0,27	3,8E-02
Ythdf3	0,30	3,8E-02
Fam13b	0,32	3,8E-02
Atxn7l2	-0,34	3,8E-02
Itih5	0,46	3,8E-02
Dennd4b	-0,28	3,9E-02
Armc5	-0,42	4,0E-02
Usp1	0,37	4,0E-02
Clasrp	-0,39	4,0E-02
Dcaf15	-0,42	4,0E-02
Egr3	-0,56	4,0E-02
Cnot3	-0,30	4,0E-02
Trmt1	-0,27	4,1E-02
Hook3	0,34	4,1E-02
Epm2aip1	0,27	4,1E-02
Cntnap1	-0,34	4,1E-02
Rnf208	-0,41	4,1E-02
Kdm4b	-0,30	4,1E-02
Fam199x	0,59	4,1E-02
Nup160	0,39	4,1E-02
Aff4	0,36	4,1E-02
Kmt2b	-0,39	4,1E-02
Cdh11	0,26	4,2E-02
Bivm	0,25	4,2E-02
Epn1	-0,29	4,2E-02
Hcn2	-0,25	4,2E-02
Arhgef4	-0,21	4,2E-02
Susd2	-0,40	4,2E-02
Fam193b	-0,34	4,2E-02
Ppp2r3a	0,31	4,2E-02
Ntn3	-0,38	4,2E-02
Prr12	-0,42	4,2E-02
Rexo1	-0,27	4,3E-02
Klhl24	0,33	4,3E-02
P4htm	-0,22	4,3E-02
Nme7	0,46	4,3E-02
Ldb1	-0,21	4,3E-02
Ssh3	-0,46	4,3E-02
Svip	0,33	4,3E-02
Ubxn11	-0,38	4,3E-02
Kdm6b	-0,47	4,3E-02
Chm	0,36	4,4E-02
Zfp326	0,26	4,4E-02

Slc45a1	-0,27	4,4E-02
Lmbrd2	1,29	4,4E-02
Kcnj12	-0,46	4,5E-02
Dcaf17	0,34	4,5E-02
Crebrf	0,37	4,6E-02
Sgce	0,27	4,6E-02
Zfyve16	0,33	4,6E-02
Flywch2	-0,49	4,6E-02
Mrpl4	-0,31	4,6E-02
Iffo1	-0,31	4,6E-02
Mfsd3	-0,39	4,6E-02
Gria2	0,44	4,6E-02
Atp13a3	0,33	4,6E-02
AI504432	0,30	4,6E-02
Sf3a2	-0,43	4,6E-02
Pold1	-0,50	4,6E-02
NA	-0,67	4,6E-02
Mov10	-0,55	4,6E-02
Vps54	0,25	4,7E-02
Mdga2	0,53	4,7E-02
Cacnb1	-0,25	4,7E-02
Rusc1	-0,32	4,7E-02
Doc2a	-0,52	4,7E-02
Lnp	0,41	4,7E-02
Zfp653	-0,30	4,7E-02
Ruvbl2	-0,34	4,7E-02
Hddc3	0,64	4,7E-02
Kcnt2	0,60	4,7E-02
Pex5	-0,20	4,7E-02
Csad	-0,33	4,7E-02
Kcnb2	0,69	4,7E-02
NA	-0,35	4,7E-02
Filip1	0,45	4,7E-02
Slitrk4	0,47	4,8E-02
Cr1l	0,38	4,8E-02
Ift74	0,32	4,8E-02
Nrgn	-0,35	4,8E-02
Abcc8	-0,41	4,8E-02
Cdk10	-0,28	4,8E-02
Zfp579	-0,44	4,8E-02
Dpyd	0,71	4,8E-02
Zfp619	0,53	4,8E-02
Tcf4	0,35	4,8E-02
Slc22a17	-0,27	4,8E-02
Uba6	0,46	4,8E-02
Pds5b	0,37	4,8E-02

2700089E24Rik	0,31	4,8E-02
Tbc1d23	0,24	4,8E-02
Rad21	0,23	4,8E-02
Htra3	-0,63	4,8E-02
Gadd45g	-0,65	4,8E-02
Agap3	-0,25	4,8E-02
Bdp1	0,34	4,9E-02
Cntrob	-0,38	4,9E-02
Dgkb	0,51	4,9E-02
Fam76b	0,26	4,9E-02
Ralgds	-0,26	4,9E-02
Akt1s1	-0,28	4,9E-02
Wfs1	-0,38	4,9E-02
NA	-0,41	4,9E-02
Mlh3	0,29	4,9E-02
Etohi1	0,57	5,0E-02
Zbtb26	0,41	5,0E-02
Acta1	-0,52	5,0E-02
Mif	-0,43	5,0E-02
Fbxl5	0,27	5,0E-02
C1galt1	0,67	5,0E-02
Telo2	-0,34	5,0E-02

Table S4: RNA-seq reveals that the transgenes coding for Psen1 and App are not differentially expressed in JQ-1 treated APP mice.

	log2FoldChange	adjusted p-value
Psen1	-0,22	0,39
App	-0,14	0,38

Table S5: RNA-seq reveals 107 differentially expressed genes completely restored in APP mice after JQ1 treatment

Gene Symbol	log2Fold Change Wtveh vs. APPveh.	adjusted p-value Wtveh. Vs. APPveh.	log2Fold Change APPveh. Vs. APPjq1	adjusted p-value APPveh vs. APPjq1	log2Fold Change Wtveh. Vs. APPjq1	adjusted p-value Wtveh. Vs. APPjq1
Smc2	-0,29	1,61E-02	0,68	4,04E-06	0,38	3,06E-02
Ccdc88a	-0,33	1,57E-02	0,59	6,08E-06	0,26	1,10E-01
Lzts2	0,43	1,28E-04	-0,45	1,13E-05	-0,03	9,28E-01
Scai	-0,58	2,91E-03	0,63	1,25E-05	0,04	9,40E-01
Zfp628	0,43	1,52E-02	-0,63	1,35E-05	-0,21	5,22E-01
Ncor2	0,47	1,74E-02	-0,67	1,98E-05	-0,21	5,16E-01
Map1s	0,41	1,29E-02	-0,62	3,11E-05	-0,22	4,49E-01
Azi1	0,28	2,23E-02	-0,54	8,22E-05	-0,27	2,22E-01
Pcsk1n	0,51	4,07E-02	-0,86	8,63E-05	-0,37	1,87E-01
Lrfn4	0,44	1,18E-02	-0,57	1,15E-04	-0,15	7,06E-01
Tia1	-0,25	8,50E-04	0,32	1,16E-04	0,06	6,77E-01
Zfp219	0,46	2,13E-02	-0,54	1,33E-04	-0,09	8,38E-01
Zfp865	0,45	1,88E-02	-0,53	1,33E-04	-0,09	8,13E-01
Nrxn2	0,33	7,88E-03	-0,39	1,49E-04	-0,07	8,06E-01
Zfp120	-0,47	2,27E-02	0,60	1,75E-04	0,11	7,92E-01
Ncln	0,29	4,34E-02	-0,41	1,97E-04	-0,12	6,25E-01
Cnot6	-0,28	4,71E-02	0,46	3,95E-04	0,17	3,08E-01
Jag2	0,36	2,32E-02	-0,46	5,30E-04	-0,11	7,03E-01
Grm4	0,54	2,76E-02	-0,71	5,38E-04	-0,18	6,87E-01
Zhx1	-0,23	3,24E-02	0,39	6,48E-04	0,15	4,06E-01
Prr7	0,58	4,17E-02	-1,01	8,47E-04	-0,44	2,47E-01
Lrp1b	-0,39	3,97E-02	0,65	1,57E-03	0,25	3,34E-01
Mroh1	0,50	2,60E-05	-0,48	1,95E-03	0,01	9,82E-01
Frs3	0,36	2,22E-02	-0,42	2,21E-03	-0,07	7,85E-01
Ppip5k2	-0,25	1,90E-02	0,32	2,71E-03	0,06	7,05E-01
Fgf14	-0,50	7,55E-04	0,54	3,01E-03	0,03	9,41E-01
Shprh	-0,24	4,09E-02	0,41	3,70E-03	0,16	3,80E-01
Usp15	-0,29	2,91E-03	0,39	4,03E-03	0,09	7,30E-01
Trdmt1	-0,41	3,60E-02	0,57	4,03E-03	0,15	5,60E-01
Zranb2	-0,25	2,04E-02	0,24	6,37E-03	-0,02	9,36E-01
Gabrg1	-0,61	2,28E-02	0,62	6,59E-03	0,00	9,97E-01
Ranbp2	-0,37	2,26E-02	0,51	6,71E-03	0,14	6,92E-01
Dhx36	-0,31	2,49E-04	0,32	8,13E-03	0,00	9,98E-01
Cic	0,51	5,83E-03	-0,46	8,47E-03	0,04	9,43E-01
Ppard	0,41	2,32E-02	-0,44	8,60E-03	-0,04	9,31E-01
Shkbp1	0,53	1,02E-02	-0,60	8,60E-03	-0,08	8,86E-01
Fem1c	-0,34	1,54E-02	0,39	8,81E-03	0,04	9,06E-01
Stx4a	0,32	5,18E-03	-0,28	9,08E-03	0,03	9,01E-01
Sf3b1	-0,17	7,53E-03	0,20	9,29E-03	0,02	8,88E-01
Tmem120	0,40	2,32E-02	-0,48	1,13E-02	-0,10	8,08E-01

b

Foxo6	0,43	4,61E-02	-0,54	1,17E-02	-0,11	7,57E-01
Xiap	-0,27	4,88E-02	0,40	1,19E-02	0,12	6,62E-01
Hiatl1	-0,26	1,08E-02	0,33	1,23E-02	0,06	8,41E-01
Grm3	-0,54	5,99E-03	0,60	1,23E-02	0,05	9,08E-01
Arhgef1	0,41	4,09E-02	-0,50	1,23E-02	-0,10	8,44E-01
Top2b	-0,27	2,01E-02	0,36	1,48E-02	0,08	7,52E-01
Bmi1	-0,26	3,95E-02	0,32	1,55E-02	0,04	8,63E-01
Ap5z1	0,40	3,12E-02	-0,47	1,56E-02	-0,09	8,40E-01
Rbm26	-0,25	2,11E-02	0,29	1,60E-02	0,03	9,04E-01
Gpm6a	-0,45	3,73E-03	0,41	1,62E-02	-0,05	9,10E-01
Whrn	0,50	1,25E-02	-0,50	1,74E-02	-0,01	9,88E-01
Cul4b	-0,37	1,36E-02	0,34	1,84E-02	-0,04	8,93E-01
Ahdc1	0,44	9,06E-03	-0,40	1,92E-02	0,04	9,05E-01
Sh3bp1	0,55	8,50E-03	-0,54	2,00E-02	-0,01	9,90E-01
Ccdc73	0,49	2,32E-02	0,55	2,07E-02	1,04	7,11E-13
Rasa1	-0,34	1,04E-02	0,38	2,08E-02	0,03	9,16E-01
Gpm6b	-0,37	2,86E-02	0,32	2,12E-02	-0,06	8,43E-01
Pcdhb14	-0,56	1,68E-04	0,55	2,15E-02	-0,02	9,78E-01
Zfp53	-0,59	4,16E-02	0,68	2,18E-02	0,09	8,77E-01
Snx13	-0,33	6,87E-04	0,31	2,18E-02	-0,03	9,15E-01
Rb1cc1	-0,34	7,27E-03	0,36	2,29E-02	0,02	9,63E-01
Zfp758	-0,59	4,05E-02	0,72	2,38E-02	0,12	8,62E-01
Tfeb	0,67	1,64E-04	-0,54	2,41E-02	0,11	8,27E-01
Zranb1	-0,34	2,45E-02	0,32	2,42E-02	-0,03	9,43E-01
Tab3	-0,29	1,06E-02	0,37	2,43E-02	0,07	8,02E-01
Kdm6a	-0,26	4,28E-02	0,33	2,43E-02	0,06	7,77E-01
Reps2	-0,39	2,70E-03	0,37	2,44E-02	-0,03	9,41E-01
Ccdc34	-0,31	2,61E-02	0,35	2,45E-02	0,03	9,21E-01
Hps4	0,59	6,03E-04	-0,44	2,48E-02	0,14	7,19E-01
Plekhh3	0,45	2,32E-02	-0,40	2,49E-02	0,03	9,53E-01
Kctd4	-0,51	5,63E-03	0,38	2,53E-02	-0,15	6,66E-01
Gltscr1	0,52	9,43E-03	-0,43	2,59E-02	0,09	8,52E-01
Abca7	0,44	3,13E-02	-0,43	2,71E-02	0,00	9,96E-01
Ccdc157	0,31	1,32E-02	-0,29	2,72E-02	0,01	9,81E-01
Scrib	0,45	1,20E-02	-0,40	2,88E-02	0,03	9,54E-01
Arfgef1	-0,24	2,54E-02	0,33	3,04E-02	0,08	7,57E-01
2310003H01Rik	0,53	5,66E-03	-0,44	3,05E-02	0,09	8,26E-01
B3galt2	-0,58	7,69E-03	0,53	3,17E-02	-0,06	9,24E-01
Ptpn23	0,36	3,89E-02	-0,39	3,19E-02	-0,03	9,44E-01
Senp7	-0,35	9,26E-03	0,35	3,19E-02	-0,01	9,84E-01
Ralgapa1	-0,26	1,25E-04	0,28	3,28E-02	0,01	9,80E-01
Fam171b	-0,41	2,99E-03	0,37	3,38E-02	-0,05	9,00E-01
Sec23a	-0,29	5,69E-03	0,29	3,46E-02	-0,01	9,82E-01
Ccne2	-0,49	2,76E-02	0,48	3,49E-02	-0,02	9,66E-01
Syne1	-0,39	1,69E-02	0,40	3,54E-02	0,01	9,82E-01

Cpeb4	-0,41	3,46E-02	0,43	3,71E-02	0,01	9,82E-01
C77370	-0,42	4,76E-02	0,49	3,71E-02	0,06	9,02E-01
Cpne9	0,87	3,40E-02	-0,92	3,79E-02	-0,06	9,48E-01
Fpgt	-0,43	2,30E-03	0,37	3,80E-02	-0,07	8,60E-01
Armc5	0,44	1,19E-02	-0,42	4,01E-02	0,01	9,85E-01
Cdh11	-0,34	3,09E-03	0,26	4,15E-02	-0,09	7,20E-01
Bivm	-0,35	1,97E-03	0,25	4,15E-02	-0,11	6,07E-01
Nme7	-0,40	1,34E-03	0,46	4,30E-02	0,05	9,04E-01
Chm	-0,47	1,25E-03	0,36	4,36E-02	-0,12	7,47E-01
Gria2	-0,56	9,22E-06	0,44	4,62E-02	-0,13	7,24E-01
Pold1	0,36	3,79E-02	-0,50	4,62E-02	-0,16	7,21E-01
AI504432	-0,36	2,55E-02	0,30	4,62E-02	-0,07	8,23E-01
Mov10	0,43	1,91E-02	-0,55	4,64E-02	-0,13	8,09E-01
Lnp	-0,35	3,62E-02	0,41	4,68E-02	0,05	9,19E-01
Kcnt2	-0,71	7,02E-03	0,60	4,69E-02	-0,11	8,44E-01
Slitrk4	-0,55	3,04E-02	0,47	4,76E-02	-0,08	8,93E-01
Zfp579	0,47	3,87E-02	-0,44	4,76E-02	0,02	9,80E-01
Gadd45g	0,59	3,36E-02	-0,65	4,80E-02	-0,07	8,91E-01
Htra3	0,71	2,30E-03	-0,63	4,80E-02	0,07	8,95E-01
Dgkb	-0,61	7,65E-03	0,51	4,87E-02	-0,11	8,38E-01
Mlh3	-0,27	4,39E-02	0,29	4,94E-02	0,02	9,38E-01

Table S6: List of primers used in this study

Primer name	Sequence
Abcc8_F	gggagatgcagagggtctc
Abcc8_R	ttctccctcgctgtctgg
Ano8_F	cttggaggaccagccaatc
Ano8_R	tgaactggaaacacctgctg
Gabra2_F	acaaaaagaggatgggcttg
Gabra2_R	tcatgacggagcctttctct
Gabrb1_F	ccctctggatgagcaaaact
Gabrb1_R	aattcgatgtcatccgtggta
Gabrg1_F	gaggcaggaagctgaaaaac
Gabrg1_R	tgctgttcatgggaatgaga
Gpm6a_F	cttggatctgcgtcagtttg
Gpm6a_R	ggaagttctcagaggcagtacaa
Gria2_F	cagtttcgcagtcaccaatg
Gria2_R	acccaaaaatcgcatagacg
Grik2_F	gcacctccaaaacctgactc
Grik2_R	catcagagcagcatcagtcg
Grik5_F	cccctcagctagcctcatct
Grik5_R	gcctcgcaccagttcttcta
Grin1_F	gctggaggagcgtgagtc
Grin1_R	agcagagccgtcacattctt
Kcnh2_F	gatcgccttctaccggaaa
Kcnh2_R	cattcttcacgggtaccaca
Kcnc3_F	gaaaccaacagggcagacc
Kcnc3_R	cagtcctcatgggccagt
Kcnt2_F	ggtgggctctattgacaacct
Kcnt2_R	cactcatggtgctttctttgtc
Kcnt1_F	gggtaaacctgggatatttgc
Kcnt1_R	ggggttgatgagtacataggaaa
Scn3a_F	gctggctacctggcattg
Scn3a_R	tgaatccacagctgcatacat
Scn9a_F	agcaggtgggacaaaggat
Scn9a_R	tctctccttggcactctttga
Lnpep_F	tcacgaactggcacatcagt
Lnpep_R	agccttcatttagccacagg
Stxbp5l_F	gacctgtaccagcgacagc
Stxbp5l_R	ccacgcatgcaaaagttgt
Bmpr2_F	gagccctcccttgacctg
Bmpr2_R	gtatcgaccccgtccaatc
Ube3a_F	cgaatggccacagcttgta
Ube3a_R	gcttcattcggctagcttca
Ncam_F	ccaagcagtggcaagagttt
Ncam_R	gccactgatcttccttgtcttta

Pcsk1n_F	ctgctcttgggccttctg
Pcsk1n_R	ggagtgctcgtctcaacca
Fcrls_F	gcctttgattgtggacatga
Fcrls_R	gatcttcagaaagtgctgggtaa
Erdr1_F	acggacggactccacaag
Erdr1_R	ggatttctgtacgcagtcagg
Vgf_F	tcccatggctgacgaaat
Vgf_R	gtggcttgactttcccagaa
Enpp2_F	tggcttacgtgacattgagg
Enpp2_R	agtgggtagggacaggaatagag
Lars2_F	ataaaggagcaagcgtccag
Lars2_R	tgggaacatggagagcaagt
Npn_F	aacaaccgtatgcccatga
Npn_R	cttcacatagatgaggctcaggat
Ubap2_F	tctcattccctgtgaaccatt
Ubap2_R	tcagtttttcaaacacaaggatg
Gria4_F	ctgccaacagttttgctgtg
Gria4_R	aaatggcaaacacccctcta
Ppp1r10_F	gagccatatgaacccattcc
Ppp1r10_R	caggtgagccaccagacc
Penk_F	agccaggactgcgctaaat
Penk_R	cagctgtccttcacattcca
Ppan_F	taaggaagtgtgtgaccccc
Ppan_R	catggaacctctcaccgcta
Mcm7_F	ccaactgggtcccatacttg
Mcm7_R	ggactacgcgatcgaaaaag
Ccnf_F	caccaggtacttgaggtggg
Ccnf_R	aaccatcttgagtctcccagaa
Brsk2_F	ttcatgccttcctgtcgat
Brsk2_R	acttgaccggcttctgga
