# Supplementary material for: The BET/BRD inhibitor JQ1 improves brain plasticity in WT and APP mice
Source: Transl Psychiatry. 2017 Sep 26;7(9):e1239–. doi: 10.1038/tp.2017.202 (PMC5639246; doi:10.1038/tp.2017.202)
Supplement: Supplementary Figures [file tp2017202x2.docx]

**Fig S1**

** Figure S1. BRD4 is displaced from the chromatin-bound fraction after JQ1 treatment. a.** The amount of BRD4 in the chromatin-bound fraction is significantly reduced after JQ1 treatment in hippocampal cells both after 30min and 24h. *** p < 0.001, 2-WAY ANOVA (F (1, 8) = 31.28), * p < 0.05, Holm-Sidak corrected t-test.

**Fig S2**

**Figure S2. Basal mobility is not affected in 8 month old APP/PS1 animals treated with JQ1. a.** 8 month old APP animals were injected with JQ1 i.p. for 3 days prior to the open field. Speed and total path travelled were not affected by JQ1 injection in APP animals. **b.** Swimming speed in the Morris Water maze was also not affected significantly in APP vehicle vs. APP JQ1-injected animals both on the first trial of the first day and on the probe test day.

**Fig S3**


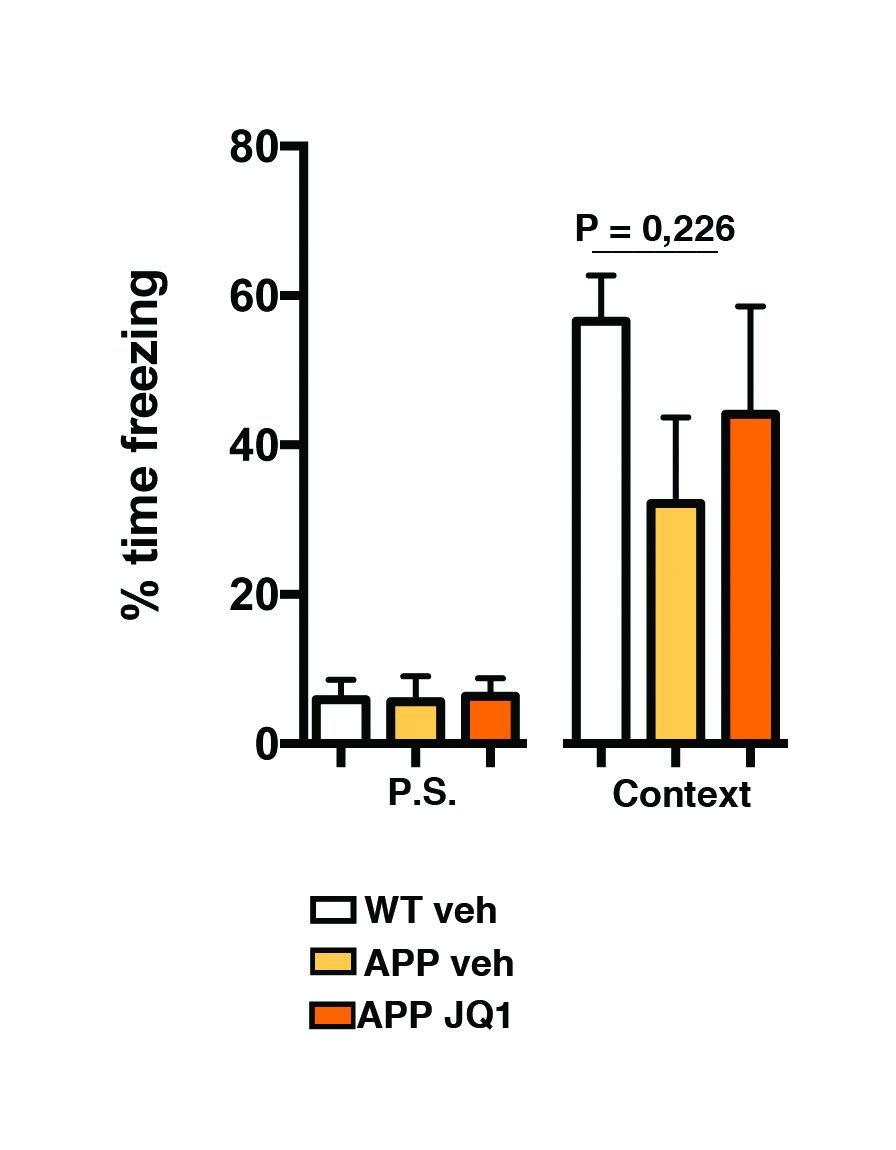


**Fig S3. Contextual fear conditioning in 8 month old APP mice.** 8 month old APP mice received daily injection in vehicle or JQ for 1 week before being subjected to contextual fear conditioning training. Vehicle treated WT mice served as an additional control. Minimal freezing behavior that was similar amongst groups was detected during the training (P.S., pre-shock). When memory was tested 24h after the training vehicle-treated APP mice showed a trend towards impaired freezing behavior, which was partially ameliorated in JQ1 treated APP mice. One-way ANOVA analysis revealed however that the difference amongst groups was not significant (P = 0.226). This data is in line with other findings showing that APP mice are more sensitive to impairments in the Morris water maze test, while fear learning is only altered at advances stages of pathology (Puzzo et al., 2014, Biochem Pharamcol, 88,4, p450; PMID 24462904). Error bars indicate SEM.

**** **Fig S4**

**Figure S4. a.** Immediate Early Gene (IEG) expression profile after acute treatment with 250nM JQ1 in hippocampal cultures. **b.** Immediate Early Gene (IEG) expression profile after acute treatment with 250nM JQ1 in hippocampal cultures. * p < 0.05, ** p < 0.01, *** p < 0.001, Holm-Sidak corrected t-test. n = 6 per group for both timepoints.

**Fig S5.**

**
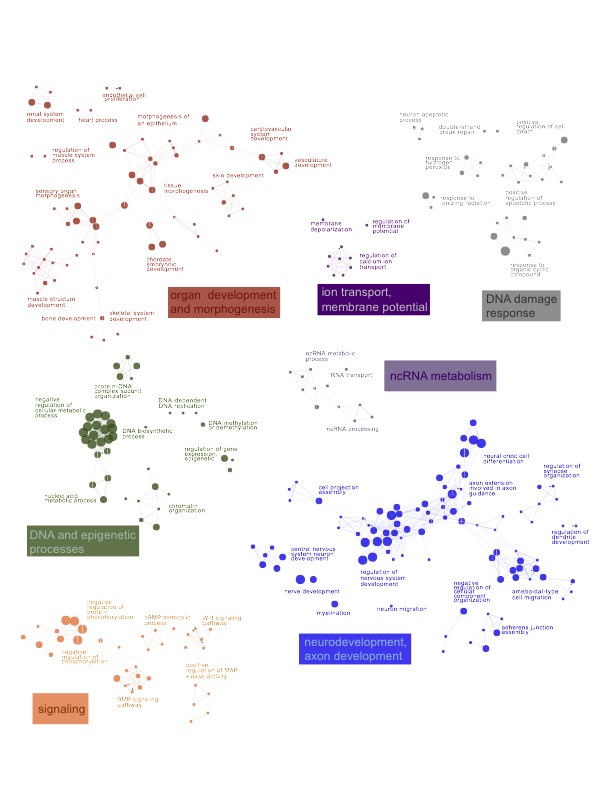
**

**Figure S5. Biological process network for genes differentially regulated in neuroblastoma cells after JQ1 treatment.** Although the model is fundamentally different from healthy hippocampal neurons in vivo, many categories are common with the ones we find in APP/PS1 vehicle vs. APP/PS1 JQ1-treated animals, including ion transport, DNA damage response and neurodevelopment.

**Fig S6.**


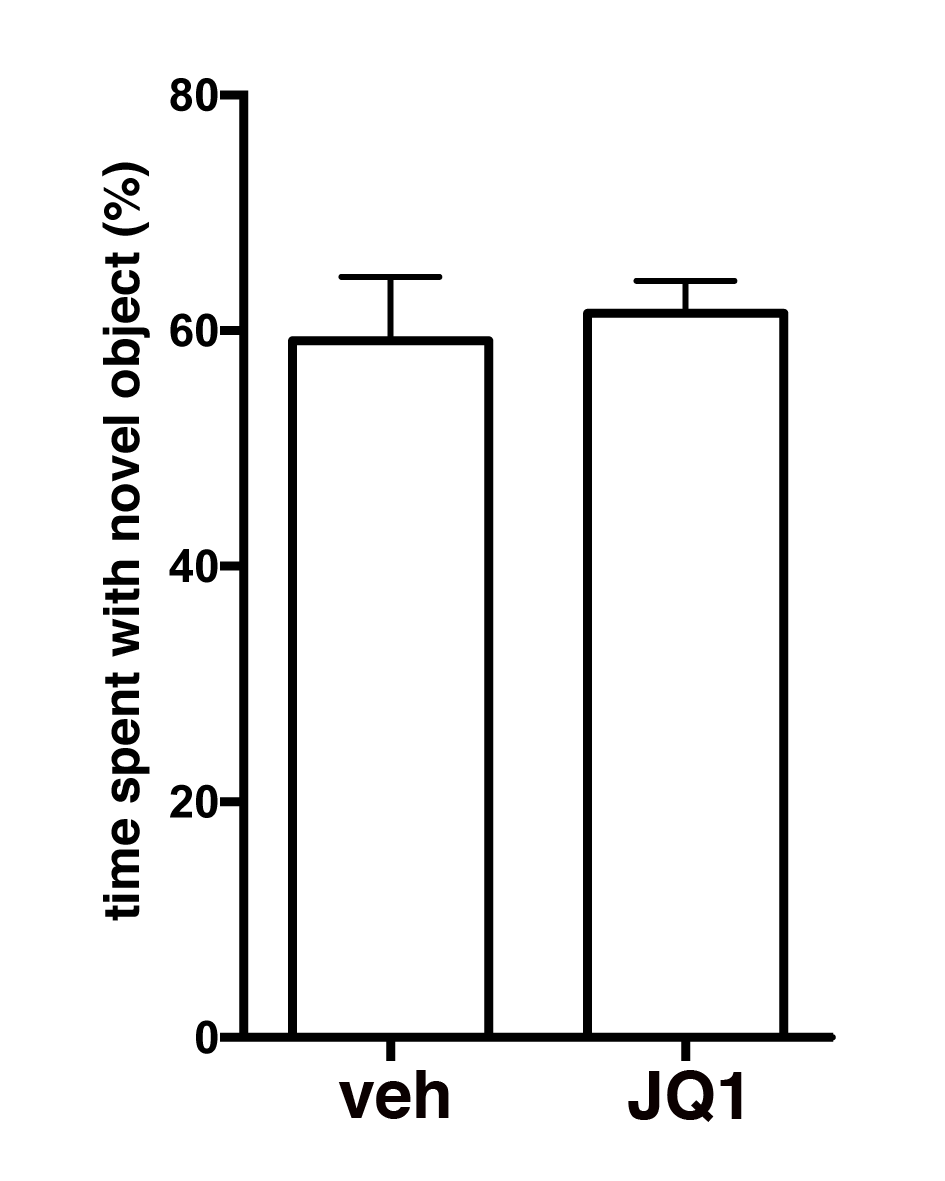


**Fig S6. Novel object recognition memory is not affected by JQ1 treatment.**

Mice received daily injection of JQ1 (intraperitoneal 50mg/kg) for 1 week (n = 9) or vehicle solution (n = 8). Before being subjected to the novel object recognition test (NOR). To this end animals were placed for 10 min in the NOR arena without any objected for habituation. On the next day animals were placed for 5 min in the same NOR arena that contained 2 identical objects. 24h later the memory test was performed by placing mice to the NOR arena with on familiar and one novel object. The time spent with the novel object was measured using the VideoMot2 System (TSE). There was no statistical difference amongst groups (t-test; *P* = 0,69). Veh; vehicle. Error bars indicate SEM.
